# Immunotherapy for pancreatic cancer: chasing the light at the end of the tunnel

**DOI:** 10.1007/s13402-021-00587-z

**Published:** 2021-03-12

**Authors:** Thomas P. Brouwer, Alexander L. Vahrmeijer, Noel F. C. C. de Miranda

**Affiliations:** 1 Department of Surgery, Leiden University Medical Center, Leiden, The Netherlands; 2 Department of Pathology, Leiden University Medical Center, Albinusdreef 2, 2333 ZA, Leiden, The Netherlands PO Box 9600, 2300 RC

**Keywords:** Pancreatic ductal adenocarcinoma, PDAC, Immunotherapy, Microenvironment, Checkpoint blockade, Stroma, combination therapies

## Abstract

**Background:**

Checkpoint blockade immunotherapy has had a significant impact on the survival of a subset of patients with advanced cancers. It has been particularly effective in immunogenic cancer types that present large numbers of somatic mutations in their genomes. To date, all conventional immunotherapies have failed to produce significant clinical benefits for patients diagnosed with pancreatic cancer, probably due to its poor immunogenic properties, including low numbers of neoantigens and highly immune-suppressive microenvironments.

**Conclusions:**

Herein, we discuss advances that have recently been made in cancer immunotherapy and the potential of this field to deliver effective treatment options for pancreatic cancer patients. Preclinical investigations, combining different types of therapies, highlight possibilities to enhance anti-tumor immunity and to generate meaningful clinical responses in pancreatic cancer patients. Results from completed and ongoing (pre)clinical trials are discussed.

## Introduction

Pancreatic ductal adenocarcinoma (PDAC) is the most common form of pancreatic cancer and the seventh leading cause of cancer deaths in developed countries, with forecasts indicating a further escalation of mortality rates in the coming decade [1, 2]. The 5-year survival rate of PDAC is 9 % and is anticipated to remain dismal for years to come [3]. Chemotherapy and surgery have been the cornerstones of PDAC treatment over the past decades. Surgical resection is the only curative option, but it is only applicable in 20% of the patients due to disseminated disease at the time of diagnosis [4]. Gemcitabine has predominantly been used over the past decades as an (adjuvant) mono-therapy with limited benefit [5]. Adjuvant combination therapies, such as the addition of capecitabine or nanoparticle albumin bound (nab)-paclitaxel to gemcitabine and 5-fluorouracil, folonic acid, irinotecan and oxaliplatin (FOLFIRINOX), have improved overall survival compared to gemcitabine monotherapy and have become established treatment options [6–8]. More recently, these combination regimens have been implemented in a neoadjuvant setting to improve the chances of successful surgical resection of locally advanced PDAC [9]. Unfortunately, such combination therapies come at the cost of significant side effects whilst overall survival remains poor, warranting new treatment avenues.

The recent breakthrough of cancer immunotherapy has had a huge impact on the outcome of patients affected by therapy-recalcitrant cancer types such as advanced melanoma and non-small cell lung cancer (NSCLC) [10–12]. Targeting the immune checkpoint molecules programmed death 1 (PD-1), programmed death ligand 1 (PD-L1), and cytotoxic T lymphocyte antigen-4 (CTLA-4) leads to reinvigoration of anti-tumor immune responses in cancer patients and, consequently, improved clinical outcomes [13–16]. So far, immune checkpoint blockade, as mono- or combination therapy, has had a limited effect in PDAC, with the exception of patients diagnosed with mismatch repair-deficient cancers [17–21]. One of the most likely explanations for the obstinate nature of PDAC with respect to immunotherapy is that this tumor type is poorly immunogenic, with at least ten times less somatic mutations compared to melanoma and lung cancer. This translates into a scarcity of mutated antigens (neoantigens) that can be targeted by the patients’ T cells [22]. Furthermore, the tumor microenvironment (TME) in PDAC plays a complex role in cancer progression and therapeutic response. Specifically, the PDAC TME is composed of a large stromal component consisting of cancer-associated fibroblasts, extracellular matrix, and immune cells with immune suppressive features that, most likely, also act as a barrier for drug delivery [23, 24].

Immunotherapy, however, may still hold potential to be used in a tailored manner for PDAC patients. Novel omics-technologies are providing more profound insights into the tumorigenic and immunologic mechanisms at play in PDAC and, as such, may support rationale-based approaches [25, 26]. In this review, we will summarize which advances in immunotherapy may translate into improved PDAC treatment options. In addition, we will discuss which combinatorial approaches are likely to maximize the potential of immunotherapy and, thus, may serve as light at the end of the tunnel for PDAC patients.

## Enhancing knowledge of PDAC immune biology to improve immunotherapy outcomes

**Fig. 1:**
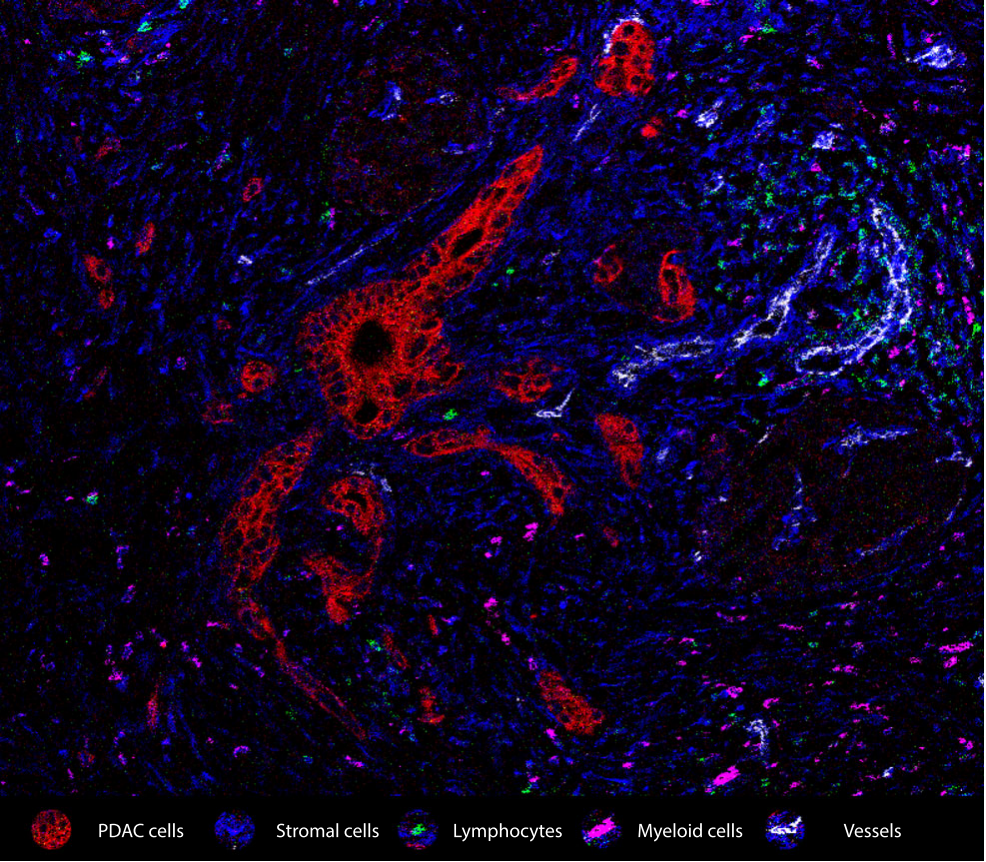
Representative image of PDAC and its microenvironment. A high stromal content (vimentin, blue) surrounds malignant pancreatic ducts (red). Lymphocytic infiltration (CD3, green) is sparse and, generally, away from the tumor cells. Myeloid cells (CD68, purple) can be abundant and often possess immune suppressive features. All these factors, together with a poor vascularization, constitute major obstacles to the success of immunotherapeutic approaches in pancreatic cancer. The image was obtained through imaging mass cytometry analysis as reported before [183]

PDAC combines a unique set of (intrinsic/extrinsic) features that makes it particularly challenging to tackle with immunotherapeutic approaches. In Fig. 1, a typical example of a PDAC TME is depicted: a large desmoplastic stroma that likely hampers drug delivery and immune cell infiltration, a low vessel density and a low abundance of immune cells. The poor immunogenicity of PDAC can, at least in part, be explained by a low mutation burden in cancer cells that fails the triggering of naturally occurring, cytotoxic immune responses [23, 27, 28].

A number of studies has focused on dissecting the various components that underlie the complex biology of PDAC. Firstly, comprehensive genomic analyses have mapped the mutational and transcriptional landscape of PDACs. Activating mutations in the *KRAS* gene are ubiquitous as they are present in over 90% of PDACs, while also inactivation of the *TP53* gene occurs frequently. Tumor suppressor genes such as *SMAD4* and *CDKN2A* are also often mutated, albeit at lower frequencies [29, 30]. Further genomic analyses have revealed a ‘long tail’ of additional recurrent mutations/alterations in genes that belong to common pathways, including RAS signaling, TGF-β signaling, WNT signaling, NOTCH signaling, cell cycle control, epigenetic regulation, and DNA damage repair, all with individual incidences below 10% [30, 31]. Tumors with a high mutation burden are uncommon in PDAC, as defects in DNA repair systems such as mismatch repair are scarce and PDAC development has not been associated with exposure to strong mutagenic carcinogens [32]. Mismatch repair deficiency was the first molecular feature to be granted a tissue-agnostic approval for cancer treatment, back in 2017, when pembrolizumab (an anti-PD-1 monoclonal antibody) was approved for the treatment of mismatch repair deficient tumors. Since then, this agent has shown an anti-tumor effect in mismatch repair deficient PDAC patients [21, 33]. DNA mismatch repair deficiency occurs in up to 1% of PDAC patients, rendering only a select group of patients eligible for anti-PD-1 therapy [34]. Of note, it has been suggested that *BRCA1*- and *BRCA2*-deficient tumors could also be susceptible to checkpoint blockade immunotherapy due to the widespread propagation of DNA damage in these tumors [32, 35]. Hereditary or somatic alterations in the *BRCA* genes have also been described in PDAC patients, but whether they confer sensitivity to checkpoint blockade immunotherapy remains to be demonstrated [36]. Beyond mutation burden, transcriptional profiling has revealed the existence of different PDAC subtypes. Bailey and colleagues revealed a subtype with immunogenic features defined by upregulation of the CTLA-4- and PD1-encoding genes, thereby identifying a subgroup of patients that could potentially benefit from checkpoint blockade immunotherapy [37]. Other works using transcriptional profiling have, however, failed to identify such a potentially immunogenic subgroup [38, 39].

In recent years, the comprehensive characterization of immune cell populations that infiltrate tumor microenvironments has been essential to understand cancer immunity as well as to devise new immunotherapeutic strategies. Cytotoxic T cells play a central role in cancer immunity and immunotherapy. Their accumulation in tumor tissues is generally associated with a better patient prognosis and a better response to checkpoint blockade immunotherapies [13, 40, 41]. Although tumor infiltration by cytotoxic T cells has been associated with an improved overall survival in PDAC, the number of cytotoxic T cells in the PDAC tumor microenvironment has been found to be significantly lower compared to cancers with a more immunogenic profile [42–44]. CD4+ T cells also play a fundamental role in the tumor immunity cycle and can differentiate into Th1, Th2, Th17 or Treg subsets. Th1 cells support anti-tumor responses through the production of cytokines such as IL-2 or IFN-γ. On the other hand, CD4+ T cells with a Th2 phenotype are sources of IL-4, IL-5 and IL-6, and induce humoral responses rather than cytotoxic ones [45]. During PDAC disease progression, a shift from predominantly Th1 to Th2 responses has been reported, thereby curtailing potential cytotoxic responses and promoting PDAC immune escape [46, 47]. Another driving force behind CD4+ T cell-mediated immune escape in PDAC are regulatory T cells (Tregs). Tregs secrete immune suppressive molecules like IL-10 and TGF-β and, therefore, their presence has generally been considered as an unfavorable prognostic factor [41, 48, 49]. However, their presence in relation to clinical outcome, as well as their immunomodulatory function in PDAC and other gastrointestinal malignancies, is still not yet fully defined. Depletion of Tregs in a preclinical PDAC model failed to relieve immunosuppression and led to accelerated tumor progression [50]. Furthermore, a systematic analysis has shown that Treg presence does not impose a negative impact on the survival of PDAC patients [51]. Myeloid cells are abundant in the PDAC tumor microenvironment and include tumor-associated macrophages and myeloid-derived suppressor cells. Tumor-associated macrophages form a highly complex and heterogeneous subset of cells that can exhibit both pro-tumorigenic as anti-tumorigenic features. In cancer, macrophages can be classified according to either their phenotype or ontogeny [52]. Phenotypically, tumor-associated macrophages are divided into M1-, classically activated, macrophages that mediate tumor regression and are associated with prolonged patient survival or M2-, alternatively activated, macrophages that support tumor outgrowth and are associated with a decreased survival. Not surprisingly, M2-macrophages are the dominant subtype in the PDAC tumor microenvironment [43, 53]. Tumor-associated macrophages defined by their ontogeny can either be monocyte/haemopoietic stem cell derived or embryonic progenitor derived [54]. In PDAC, monocyte-derived tumor-associated macrophages play a more prominent role in antigen presentation, whilst embryonically derived tumor-associated macrophages, the most frequent subtype, exhibit a pro-fibrotic transcriptional profile and support tumor progression [55]. Interestingly, the subtyping based on ontogeny has led to the discovery of CSFR1 and CXCR4 as potential immunotherapeutic targets to specifically deal with embryonic progenitor-derived tumor-associated macrophages [56, 57]. Myeloid-derived suppressor cells (MDSCs) form another heterogeneous population of (immature) myeloid cells that can also be subdivided into two main subsets: a monocytic subset and a granulocytic subset, the latter being most prominent in the PDAC tumor microenvironment [58]. Both subsets of myeloid-derived suppressor cells actively suppress host immunity through a variety of mechanisms, including the production of arginase and nitric oxide synthase (NOS), with the abundancy of myeloid-derived suppressor cells being linked to progressive disease [59, 60]. In addition, myeloid-derived suppressor cells can amplify the immune suppressive activity of tumor-associated macrophages and dendritic cells via molecular cross-talk [61]. Dendritic cell activation in PDAC has, for example, been found to be inhibited by the production of nitric oxide synthase from myeloid-derived suppressor cells [62]. Dendritic cells are professional antigen presenting cells that are vital for the mounting of anti-tumor adaptive immune cell responses. During PDAC progression, tumor-derived cytokines can induce dendritic cell tolerogenic phenotypes and, thereby, lose their antigen presenting capacity curtailing the development of a competent anti-tumor immune response [63, 64].

Lastly, the large stromal compartment in PDAC consists of both cellular and non-cellular components that play pivotal roles in malignant cell growth and, possibly, in hampering drug delivery and immune cell infiltration [65, 66]. Crosstalk between tumor cells and its microenvironment, i.e., with cancer-associated fibroblasts (CAFs), pancreatic stellate cells, and immune suppressive cells can lead to the onset of desmoplasia, a profoundly altered stroma with pronounced fibrosis and a shift in composition of the extracellular matrix.[67, 68]. The particular stromal and fibrotic character of PDAC is considered to be a major pillar that supports the aggressive nature of PDAC, including tumor cell proliferation, immune escape, angiogenesis and metastasis. Interfering with stromal accumulation may be a potential approach to improve the outcomes of other therapeutic interventions [39, 69].

In summary, the development of immunotherapies that are efficient for PDAC patients should account for the idiosyncrasies of this disease and consider aspects from cancer genetics, immune cell composition and the role that the stromal compartment plays in this disease.

## State of the art immunotherapies in pancreatic cancer

### Immune checkpoint blockade therapies

To date, targeting CTLA-4 and the PD-1/PD-L1 axis with immune checkpoint blockers (ICB) has, with the exception of patients diagnosed with mismatch repair-deficient tumors, failed to make an impact in PDAC (NCT02558894, NCT00729664, NCT00112580, NCT01295827, NCT01876511*,* see Table 1 for information concerning published clinical trials using immunotherapy including NCT-numbers) [17–21]. On the other hand, multiple studies have indicated that high expression of PD-1/PD-L1 or CTLA-4 in the PDAC tumor microenvironment is associated with a worse outcome, suggesting that targeting CTLA-4 or the PD-1/PD-L1 interaction may yield therapeutic benefit [70–72]. Combining immune checkpoint blockers with chemotherapy (platinum-based, anti-metabolite or anthracyclines) has led to encouraging results in several clinical studies in other cancer types and may, therefore, also constitute a viable strategy for PDAC [73–75]. DNA damaging agents can contribute to the cancer immunity cycle in several ways: they can cause immunogenic cell death and thereby increase antigen availability while supplying inflammatory signals, they can augment the activity of antigen-presenting cells and, finally, they can alleviate immunosuppression by depleting immune cell subsets such as myeloid-derived suppressor cells or Tregs [76–80]. Gemcitabine, the most commonly used chemotherapeutical compound in PDAC, has been shown to increase antigen presentation and to reprogram tumor-associated macrophages towards an inflammatory phenotype [76, 81]. Platinum-based compounds, such as oxaliplatin, mainly impair DNA synthesis which ultimately drives immunogenic cell death providing molecular signals that activate antigen presenting cells [82, 83]. Taxanes, such as nab-paclitaxel, do not appear to lead to immunogenic cell death, but their chemo-modulatory effects have been shown to enhance cytotoxic T cell-mediated tumor cell killing [84]. For PDAC, two phase I trials (NCT00556023, NCT02331251) have been conducted where gemcitabine was combined with either tremelimumab (anti-CTLA-4) or pembrolizumab (anti-PD-1), and both approaches resulted in stabilization of disease for most patients, with two patients demonstrating partial responses in the tremelimumab study [85, 86]. A subsequent phase II trial (NCT02331251), using gemcitabine with nab-paclitaxel and pembrolizumab (anti-PD-1) did, however, not meet its primary endpoint of > 15% complete response rate [87]

**Table 1:** Published clinical trials using (combinatorial) immunotherapy in PDAC patients

Author	Type of immunotherapy	Phase trial	NTC-numbers
	*Checkpoint blockade as monotherapy or double therapy with or without chemo- or radiotherapy*		
O'Reilly *et al.* [17]	anti-PD-1 (Durvalumab) ± anti-CTLA-4 (Tremelimumab)	II	NCT02558894
Brahmer *et al.* [18]	anti-PD-L1	I	NCT00729664
Royal *et al.* [19]	anti-CTLA-4 (Ipilimumab)	II	NCT00112580
Patnaik *et al.* [20]	anti-PD-1 (Pembrolizumab (MK-3475))	I	NCT01295827
Le *et al.* [21]	anti-PD-1	II	NCT01876511
Aglietta *et al.* [85]	anti-CTLA-4 (Tremelimumab (CP-675,206)) ± gemcitabine	I	NCT00556023
Weiss *et al.* [86]	anti-PD-1 (Pembrolizumab) + Gemcitabine (± Docetaxel or nab-Paclitaxel or Vinorelbine or Irinotecan or Liposomal Doxorubicin)	Ib	NCT02331251
Weiss *et al.* [87]	anti-PD-1 (Pembrolizumab) + Gemcitabine + nab-Paclitaxel	Ib/II	NCT02331251
	*Cancer vaccines*		
Lutz *et al.* [103]	GVAX	II	NCT00084383
Le *et al.* (2010)	GVAX + CRS207	Ib	NCT01417000
Le *et al.* [107]	GVAX + CRS207	IIb	NCT02004262
Le *et al*. [109]	anti-CTLA-4 (Ipilimumab) + GVAX	Ib	NCT00836407
Sonntag *et al.* [114]	Neoepitope-derived multipeptide vaccines	N/A	
Gjertsen *et al*. [116]	RAS peptide vaccination	I	CTN RAS 95002
Suzuki *et al*. [118]	KIF20A/VEGFR1/VEGFR2 peptide vaccination	II	
Miyazawa *et al.* [119]	KIF20A/VEGFR1/VEGFR2 peptide vaccination	II	
Middleton *et al.* [112]	Telomerase peptide vaccine GV1001	III	ISRCTN4382138
	*Oncolytic viruses*		
Mulvihill *et al*. [128]	Adenovirus (ONYX-015)	I	
Hecht *et al.* [129]	Adenovirus (ONYX-015)	I/II	
Nakao *et al.* [130]	Oncolytic virus (HF10 )	I	
Hirooka *et al*. [131]	Oncolytic virus (HF10 )	I	
	*Adoptive T cell therapies*		
Thistlethwaite *et al.* [149]	Carcinoembryonic Antigen (CEACAM5)-specific CAR T cells	I	
Beatty *et al.* [150]	Mesothelin-Specific Chimeric Antigen Receptor T Cells	I	
	*CD40 antibodies*		
Beatty *et al.* [158]	CD40 monoclonal antibody (CP-870,893)	I	NCT00711191
O'Hara *et al.* [159]	CD40 monoclonal antibody (APX005M) ± anti-PD-1 (Nivolumab)	I	NCT02482168
	*Targeting the stromal barrier*		
Melisi *et al.* [168]	TGF-β Receptor I Kinase inhibitor (Galunisertib) + anti-PD-L1 (Durvalumab)	Ib	NCT02734160
Richeldi *et al.* [174]	anti-Connective Tissue Growth Factor (Pamrevlumab)	II	NCT01890265
Hingorani *et al.* [178]	PEGylated Recombinant Human Hyaluronidase	Ib	NCT01453153
Hingorani *et al.* [179]	PEGylated Recombinant Human Hyaluronidase	II	NCT01839487
Ramanathan *et al.* [180]	PEGylated Recombinant Human Hyaluronidase	Ib/II	NCT01959139

Radiotherapy is another widely used treatment modality. Based on (pre)clinical data, radiotherapy causes damage to both cancer cells and stromal cells and can trigger conversion of the tumor microenvironment from a “cold” to a “hot” immunological state [88–91]. In support of this, a phase I clinical melanoma trial has shown that radiotherapy can sensitize tumors to immune checkpoint therapy [92]. Clinical studies on combination strategies including radiation are rapidly progressing, with several studies also addressing abscopal effects of this treatment modality that may favor immunotherapy [93–95]. Currently, a phase II trial is ongoing with patients diagnosed with metastatic pancreatic cancer to determine the efficacy and safety of nivolumab or nivolumab plus ipilimumab administered concurrently with high dose radiotherapy (NCT02866383). Additional clinical studies are currently enrolling patients to address the most important issues of radiotherapy: dosing and timing.

### Vaccination strategies

Cancer vaccines have successfully been introduced as therapeutic measures for prostate cancer, cervical cancer and premalignant vulvar intraepithelial neoplasia [96–98]. In PDAC, the development of an immunotherapeutic vaccine is faced with the challenge of identification of a suitable cancer antigen, taking into account both its specificity for the malignant tissue and its immunogenic potential [99]. To date, different vaccination strategies have been exploited for cancer treatment: heterologous whole-cell vaccines, autologous cellular vaccines and antigen-based vaccines (DNA-, RNA- or protein-based).

The most extensively clinically tested cancer vaccine in PDAC is GVAX, a heterologous whole-cell vaccine composed of two irradiated allogeneic PDAC cell lines modified to secrete GM-CSF [100]. The rationale behind this vaccine is that the cell lines that constitute the vaccine provide a representative source of PDAC antigens, eliminating the need for personalized approaches. The inclusion of GM-CSF is aimed at inducing the recruitment of immune cells, including effector T cells [101, 102]. In a phase II study (NCT00084383), 60 patients treated with adjuvant chemotherapy in combination with GVAX showed 17.3 months disease-free survival and 24.8 months overall survival [103]. Although there was no significant survival benefit compared to surgery followed by chemoradiation (median survival 20.3 months), GVAX induced the expansion of mesothelin-specific CD8+ T cells. Mesothelin is considered to be an attractive tumor antigen in that it has a very limited expression profile in healthy tissues, with the exception of cells lining pleura, peritoneum and pericardium, but a high expression in PDAC cells. Furthermore, mesothelin is thought to be involved in PDAC progression and metastasis [104, 105]. Subsequently, CRS207, a live-attenuated listeria vaccine engineered to express mesothelin was added to GVAX and showed promise in a metastatic setting when compared to GVAX alone (6.1 vs 3.9 months overall survival) (NCT01417000) [106]. Unfortunately a phase II trial (NCT02004262) did not reveal a superior effect of CRS207-GVAX vaccination compared to chemotherapy [107]. In spite of both phase II trials failing to show superior effects compared to standard chemotherapeutic therapies, the GVAX vaccination did induce an adaptive immune response against PDAC, thereby demonstrating a potential to convert a non-immunogenic tumor microenvironment into an immunogenic one, and putatively turning PDAC patients into better candidates for immune checkpoint blockade therapy. In line with these notions, anti-PD-1 therapy in combination with GVAX was found to improve overall survival compared to the respective monotherapies in preclinical models [108]. The PD-1 blockade enhanced the vaccine-induced anti-tumor immune responses by increasing CD8+ T cell infiltrates and promoting tumor-specific interferon-γ production by CD8+ T cells in the tumor microenvironment. The possible synergism between GVAX vaccination and immune checkpoint blockers in a clinical setting was confirmed in a phase Ib trial (NCT00836407) with patients receiving the combination of GVAX and an anti-CTLA-4 antibody (ipilimumab). In a cohort of locally advanced or metastatic PDAC patients, the combined treatment group showed a longer overall survival (median 5.7 months) than the group treated with ipilimumab alone (3.6 months) [109]. The success of this combinatorial setting was proposed to be caused by a GVAX-induced expansion of mesothelin-specific CD8+ T cells within the tumor microenvironment, thereby enhancing the efficacy of the anti-CTLA-4 antibody treatment. Several trials are currently ongoing, combining GVAX and anti-PD-1 antibody treatment (NCT02451982; NCT02648282). These trials are expected to provide further evidence for this combinatorial approach.

Antigen-targeted vaccination employs another strategy aimed at stimulating an immune response specifically directed against one or multiple cancer antigens. Neoantigens are considered to be truly tumor-specific, as they arise from somatic mutations in cancer cells [110]. Several neoantigens can be targeted simultaneously per patient, provided that they are expressed in cancer tissues, presented in complex with HLA molecules and, ideally, clonal [111, 112]. Although PDACs exhibit considerably less somatic variation than other immunogenic cancer types, neoantigen recognition by T cells has been demonstrated in PDAC patients, which would theoretically allow the development of specific immunotherapies [113]. So far, only one case report has been published on the use of neoantigen vaccination of a PDAC patient in a clinical setting. The personalized neoantigen vaccine consisted of four peptides derived from two mutated proteins (RIM1 and KIF4B) that were determined as optimal binders to the patient’s HLA alleles [114]. Three of those four peptides were shown to elicit reactivity by various CD4+ T cell clones. The patient remained in complete remission for more than four years after several rounds of therapeutic vaccination combined with GM-CSF and FOLFIRINOX in parallel. This case report demonstrates the capacity of a neoantigen vaccine to produce an immune response that might contribute to a prolonged clinical remission, warranting further investigation in clinical trials of the capabilities of neo-epitopes to generate measurable neoantigen-specific CD4 and CD8 T cell responses (NCT03956056).

Antigens derived from mutations in driver genes are optimal targets for therapeutic vaccination. Mutations at driver genes are more likely to be clonal and they are often shared between patients, albeit that the number of mutations targeting driver genes is generally much lower than those targeting passenger genes in any given tumor [22]. Common mutation targets in PDAC include *KRAS*, *TP53*, *SMAD4* and *CDKN2A*, with *KRAS* mutations occurring in over 90% of PDAC patients, thereby constituting an attractive target for neoantigen-based vaccines [115]. Targeting *KRAS* mutant protein with a single- or poly-peptide vaccine in combination with GM-CSF has been shown to induce antigen-specific T cell responses in a phase I/II clinical trial, but it remained unclear whether those T cells actually recognized and killed tumor cells [116]. Long-term follow up of 23 patients in the previous study showed long term immunological memory against the KRAS antigens and a 20% 10-year survival rate (4/20 patients) [117]. Building on these results, a phase I study is currently recruiting PDAC patients to evaluate the safety and immune response to a pooled mutant-*KRAS* peptide vaccine with poly-ICLC adjuvant in combination with nivolumab and ipilimumab after adjuvant standard of care treatment (NCT04117087).

Other clinical trials with peptide-based vaccines using tumor-associated antigens have yielded mixed results. Tumor-associated antigens are not derived from mutations, but can be highly expressed in PDAC cells. A peptide cocktail vaccine, OCV-C01, containing peptides from the vascular endothelial growth factor receptor (VEGFR) 1 and VEGFR 2 proteins, both associated with neovascularization, and a kinesin-family protein (KIF20A)-derived peptide, combined with chemotherapy was tested in an adjuvant setting. This peptide cocktail vaccine showed no benefit in 1-year survival, overall survival or progression-free survival rates, but did improve disease-free survival in per-protocol analysis and led to the generation of antigen-specific cytotoxic T-cell responses in PDAC patients [118, 119]. Human telomerase reverse transcriptase catalytic subunit (hTERT) is a component of the human telomerase machinery and a rate limiting determinant of its enzymatic activity [120]. Its expression has been found to be upregulated in several cancers including PDAC. GV1001, a human telomerase reverse transcriptase catalytic subunit-derived peptide vaccine showed promising results in a phase I/II dose escalation study, demonstrating antigen-specific T cell responses and prolonged patient survival [121]. Unfortunately, the addition of GV1001 in combination with GM-CSF, gemcitabine and capecitabine in a phase III trial failed to improve the overall survival of PDAC patients [122].

In conclusion, PDAC cancer vaccination strategies have shown mixed clinical results. Although most cancer vaccines tested induced vaccine-specific T cell responses, it remains to be determined how often the targeted antigens are actually being presented by tumor cells, particularly in the context of the generation of cytotoxic T cell responses. If those antigens are indeed presented, vaccination approaches could be important complements to immune checkpoint blockade therapies.

### Oncolytic viral therapies

In recent years, there has been a renewed interest in oncolytic viral (OV) therapy as an anti-cancer treatment modality. This approach makes use of replication-competent viruses, which replicate within the host, and preferentially target and lyse tumor cells, thereby potentially inducing robust and long lasting immunity [123]. Moreover, since the first FDA-approved oncolytic viral therapy, T-VEC (Imlygic™), a recombinant human herpes simplex virus type 1 (HSV-1), oncolytic viruses have been engineered for optimization of tumor selectivity and enhanced immune stimulation [124]. Oncolytic viruses for the treatment of pancreatic cancer that have been tested in recent experimental and clinical studies include adenoviruses, herpesviruses and reoviruses [125]. Oncolytic adenoviruses were among the earliest to enter clinical trials. ONYX-015 is an E1B 55-kDa gene-deleted adenovirus that can replicate in and lyse p53-deficient cancer cells, but not cells with functional p53 [126, 127]. A dose-escalation phase I study showed that intra-tumoral injection of ONYX-015 into primary pancreatic cancers was feasible and well tolerated, but without objective responses and absence of viral replication in biopsy specimens [128]. A subsequent phase II trial administrating ONYX-015 in combination with gemcitabine in unresectable primary pancreatic tumors showed partial tumor regression in two patients, whereas two patients showed minor responses, six patients showed stable disease and 11 patients showed tumor progression [129]. HF10, which is a naturally mutated virus, has a replication capacity similar to or higher than wild-type HSV-1 in most types of transformed cells. Nakao *et al*, performed a phase I clinical trial and treated six PDAC patients with HF10. After treatment three patients were stable, one was in regression and two showed disease progression [130]. A more recent study showed that directly injected HF10 under endoscopic ultrasound guidance in combination with erlotinib and gemcitabine administration, was safe and had an anti-tumor effect. Out of nine patients who completed the treatment, three showed partial responses, four showed stable disease and two experienced disease progression [131]. Other oncolytic viruses include wild type rat parvovirus H1 (ParvOryx) and a variant of a reovirus (Reolysin). These have been extensively tested in PDAC patients, showing good tolerability but no superior effect on survival [132–134]. To increase their anti-cancer potency, oncolytic viruses have been modified to express checkpoint inhibitory antibodies or immune stimulatory molecules, thereby directing their expression in tumor tissues whilst avoiding adverse effects resulting from the systemic delivery of immune modulators [135–138]. Despite several viruses that have been tested in PDAC, their clinical efficacy was limited. If oncolytic viral PDAC therapies are to be considered, further studies are urgently needed to enable the translation of these potentially successful therapies into clinical use.

### Adoptive T cell therapies

Adoptive T cell therapy (ACT) involves the administration of autologous immune cells that can be enriched for tumor specificity, to cancer patients [139]. To achieve this, adoptive T cell therapy can make use of a patient’s naturally occurring tumor-infiltrating lymphocytes (TILs) or tumor-specific T cells obtained from peripheral blood-derived lymphocytes, which can be expanded and activated ex vivo by incubation with autologous tumor material or cancer antigens [140]. A central requirement for the efficacy of this approach is the existence of cancer antigen-specific T cells. These are, however, often not present or difficult to isolate from most PDAC patients [141]. In the absence of naturally occurring tumor-specific T cells, strategies that make use of T cell engineering may be an alternative.

Chimeric antigen receptor (CAR) T cell therapy is an adoptive T cell strategy that makes use of T cells genetically engineered to express chimeric antigen receptors (CARs). CARs are composed of an extracellular, antigen-binding domain (typically corresponding to the variable regions of an antibody) and an intracellular TCR-signaling domain. Compared to T-cell receptor therapy, CAR T cells are not HLA-restricted and can potentially be engineered to recognize any target expressed on the surface of tumor cells [142]. In B cell malignancies, CAR T cell therapies have shown impressive results, as they target B cell lineage-specific molecules such as CD19, CD20 and CD22 that are not expressed in other tissues [143]. The effectiveness of CAR T cell therapies in solid tumors has so far been limited [144]. PDAC, like other solid tumors, does not present ideal CAR targets, as there is a paucity of cell surface tumor-specific molecules. Besides, the profound degree of immunosuppression limits both the persistence of CAR T cells and their ability to effectively traffic to tumor sites [145]. Preclinical PDAC studies using genetically engineered CARs expressing tumor-associated antigens, including CEA, Her2 and MUC-1 showed promising results [146–148]. Unfortunately, a feasibility study in patients with advanced CEACAM5^+^ malignancies, including one PDAC patient, using anti-CEA CAR T cell therapy provoked significant respiratory toxicities, resulting in premature closure of the study [149]. On the other hand, a phase I trial has been completed using CAR T cells targeting mesothelin in chemotherapy-refractory metastatic pancreatic cancer patients and showed preliminary antitumor efficacy [150]. Local administration of CAR T cells by intra-tumoral injection or the use of biopolymer scaffolds could lead to superior antitumor responses in solid tumors [151]. To augment their specificity and decrease their off-target toxicity, a new generation of CARs is under investigation where CAR T cells are equipped with the ability to produce immune modulatory cytokines or checkpoint inhibitors [152]. Interestingly, the addition of anti-PD-1/PD-L1, in preclinical models restored the killing capacity of CAR T cells and diminished the amount of immune suppressive cells and the exhaustion of T cells [153, 154]. A wider therapeutic application of CAR T cells in solid tumors holds promise if the delivery to tumor sites and the persistence of CAR T cells can be improved.

### CD40 agonists

During the process of T cell priming, the presence of CD40 ligand (CD154), expressed by helper T cells, is essential for competent activation of dendritic cells and subsequent antigen presentation and provision of co-stimulatory signals to cytotoxic T cells [155]. CD40 agonists can, therefore, bypass the potential absence of T helper signaling in less immunogenic cancer types such as PDAC and license antigen presenting cells to promote T cell activation [156]. Preclinical testing of a CD40 agonist together with gemcitabine significantly reduced PDAC tumor burden and improved survival in mice, compared to monotherapy with either drug [157]. A phase I clinical trial (NCT00711191) using CD40 agonist monoclonal antibody therapy in combination with gemcitabine showed an objective response of 19% in patients with advanced PDAC, with immune activation [158]. A subsequent phase Ib trial (NCT02482168) with metastatic pancreatic cancer patients treated with chemotherapy and a CD40 agonist with or without an anti-PD1 antibody (nivolumab) showed promise with definitive results expected soon [159].

## Targeting the stromal barrier to guide immunotherapy

An avenue worth pursuing to improve the success of immunotherapy in PDAC patients is the breaking down of the stromal desmoplastic barrier and the targeting of cancer-associated fibroblasts. High desmoplasia with low vascular perfusion potentially impedes the delivery of drugs as well as immune cell infiltration. Furthermore, it contributes to a hypoxic environment which, in turn, promotes the accumulation of immunosuppressive cells and aggressive features of tumor cells [23]. Several molecules are involved in the buildup and maintenance of a pronounced PDAC stromal component and, if targetable, they may sensitize PDAC to other treatment regimens, including immunotherapy.

### Focal adhesion kinase (FAK)

Focal adhesion kinase (FAK), a non-receptor protein tyrosine kinase, is a mechano-sensor that is crucial for crosstalk between different components of the extracellular matrix and is expressed by almost all tissues and cell types [160, 161]. In PDAC patients, FAK is activated and overexpressed in the neoplastic cells and, to a lesser extent, in the stromal cells within the tumor microenvironment. Its expression is associated with a poor prognosis [162]. Preclinical testing of FAK inhibition resulted in reduced fibrosis in tumors and a decreased proliferation of cancer cells [163]. Intriguingly, additional preclinical work using FAK inhibition in combination with PD-1 blockade revealed increased amounts of tumor-infiltrating cytotoxic T cells, reductions in tumor burden and an improved survival [162]. Currently, two phase II clinical trials are ongoing enrolling pancreatic and other solid cancer patients receiving Defactinib (Verastem Oncology) in combination with an anti-PD-1 monoclonal antibody (Pembrolizumab) (NCT02758587, NCT03727880).

### Transforming growth factor β (TGF-β)

Transforming growth factor β, with its different isoforms, *TGFB1*, *TGFB2* and *TGFB3*, represents a family of cytokines that are ubiquitously expressed during tumorigenesis and have pleiotropic effects [164, 165]. In the desmoplastic stromal reaction of PDAC, TGF-β signaling, specifically TGFβ receptor I (TGFβRI), acts as an activator of pancreatic stellate cells and induces/maintains its own expression through a positive feedback loop, thereby supporting fibrosis and immune evasion [166]. A preclinical co-culture model using Galunisertib (LY2157299 monohydrate), an oral small molecule inhibitor of the chimeric TGFβRI-ALK5 kinase, revealed that this feedback loop can be blocked [167]. Subsequent clinical testing in a PDAC phase Ib trial, comparing Galunisertib in combination with gemcitabine versus gemcitabine alone, showed marginal results with an improved overall survival in the Galunisertib cohort (8.9 vs 7.1 months) [168]. A phase Ib trial (NCT02734160) looking into the synergy of Galunsertib and an anti-PD-L1 inhibitor (Durvalumab) resulted in one partial response and 7 out of 32 patient showing stable disease (disease control rate 25%), warranting further consideration for PDAC patients [169].

### Connective tissue growth factor

Connective tissue growth factor (CTGF/CCN2) is a member of the CCN family of proteins and is involved in extracellular matrix production, desmoplasia, tumor cell proliferation, adhesion, migration, angiogenesis and metastasis [170]. CTGF is overexpressed in human pancreatic cancer due to activation of the RAS/MAP-ERK pathway, which occurs in the vast majority of PDACs [171, 172]. Using a mouse model, inhibition of CTGF was shown to decrease tumor growth without attenuation of the chemotherapeutic effects of gemcitabine [173]. Pamrevlumab (Fibrogen), an anti-CTGF therapeutic, was tested in a phase II trial (NCT01890265) in patients with idiopathic pulmonary fibrosis, another disease exhibiting CTGF overexpression [174]. After showing promising results, a phase III study is now ongoing also enrolling pancreatic cancer patients (NCT03941093).

### PEGylated human recombinant hyaluronidase

Hyaluronic acid (HA) is another potential target within the pancreatic cancer stroma. Hyaluronic acid is a hydrophilic glycosaminoglycan whose production leads to increased interstitial tumor pressure, thereby limiting the access of potentially effective circulating anticancer drugs via reduced tumor perfusion [175]. In a retrospective analysis, pancreatic cancer patients with a high hyaluronic acid accumulation exhibited a median overall survival of 9.3 months, compared with 24.3 months for those with a low accumulation [176]. In a preclinical PDAC model, enzymatic depletion of HA resulted in increased tumor perfusion allowing other chemotherapeutic drugs to penetrate the tumor microenvironment, thereby decreasing the tumor burden and improving survival [177]. A subsequent phase Ib/II multicenter randomized placebo-controlled study (NCT01453153) was conducted evaluating PEGPH20 combined with gemcitabine in patients with previously untreated stage IV PDAC [178]. The addition of PEGPH20 to gemcitabine was especially beneficial in patients with HA-high tumors, showing higher response and longer progression-free survival rates. To improve the chemo-sensitivity of the PDAC patients, two subsequent trials (NCT01839487, NCT01959139) tested the combination of PEGPH20 with gemcitabine and nab-paclitaxel [179] or FOLFIRINOX [180]. Interestingly, the phase II trial using gemcitabine plus nab-paclitaxel chemotherapy showed an improvement in progression free survival when PEGPH20 was added to HA-high tumors [179]. The addition of PEGPH20 to FOLFIRINOX had, however, a detrimental effect in PDAC patients [180]. Several clinical trials are currently ongoing, combining PEGPH20 with chemotherapy, checkpoint blockade therapy, radiation therapy and rivaroxaban in patients with stage IV pancreatic cancer.

## Conclusions and perspectives

Immunotherapy for advanced PDAC desperately needs a breakthrough. To maximize success rates of immunotherapy in an era of personalized medicine, molecular and immune profiling should be the starting points of treatment selection. Recent classifications of PDAC on the basis of molecular subtypes related to immunogenicity could aid in immunotherapeutic treatment selection, although its clinical implementation still needs to be validated [37]. Emerging genomic and other biomarker profiles have provided unprecedented opportunities to identify novel targets and strategies establish personalized therapeutic approaches. The advent of multidimensional single-cell technologies that include spatial information will shed light on the tremendous cellular diversity that exists within PDACs as well as heterogeneity across PDAC patients. Novel (checkpoint) targets, such as VISTA, CSFR-1 and CCR5, have already been identified, and some of them are currently being tested in a clinical setting [44]. Although novel (checkpoint) targets are anticipated, we should first build upon what has already been shown in clinical settings. For PDAC, it seems to be particularly important to provide an inflammatory trigger that initiates, or re-initiates, anti-tumor responses. New treatment strategies should incorporate an immune stimulatory approach before making use of conventional/novel checkpoint blockade therapies. Of note, advances in the identification and targeting of neoantigens may become a safe and effective way to trigger anti-tumor immune responses [181, 182]. Currently, other agents that stimulate T cell activation and priming, CD40 agonists in particular, seem most promising in providing an immune trigger in PDAC patients. Its putative synergy with checkpoint blockade therapy is currently being tested and will hopefully hold future promise (see Table 2 for noteworthy trials). In addition, it may be particularly important, and useful, to target specific components of the stromal compartment in PDAC, thereby facilitating the infiltration of immune cells as well as other therapeutic agents. Pamrevlumab (Fibrogen), an anti-CTGF therapy, has already moved to phase III testing and, as such, may provide a new window of opportunityr not only for immunotherapy but also for conventional chemotherapies.

**Table 2:** Notable ongoing clinical trials using (combinatorial) immunotherapy in PDAC patients

Type of immunotherapy	Phase trial	NTC-numbers
Gemcitabine + Nab-paclitaxel + CD40-agonist (APX005M) +/- anti-PD-1 (Nivolumab)	Ib/II	NCT03214250
Anti-PD-1 (Pembrolizumab) + CD40-agonist (CDX-1140) + FLT3 Ligand (CDX-301)	I	NCT03329950
Radiotherapy + anti-CTLA-4 (Ipilimumab) + anti-PD-1 (Nivolumab)	I	NCT02866383
SBRT + Anti-PD-1 (Nivolumab) + anti-CSF1R (Cabiralizumab)	II	NCT03599362
SBRT + anti-CTLA-4 (Tremelimumab) + anti-PD-1 (Durvalumab)	I/II	NCT02311361
SBRT + GVAX + anti-PD-1 (Nivolumab) + anti-CCR2/CCR5	I/II	NCT03767582
Cyclophosphamide + GVAX +/- anti-PD-1 (Nivolumab) + anti-CD137 (Urelumab)	I/II	NCT02451982
Cyclophosphamide + GVAX + anti-PD-1 (Pembrolizumab) + SBRT	II	NCT02648282
Cyclophosphamide + GVAX + anti-PD-1 (Pembrolizumab) + anti-CSF1R (IMC-CS4)	I	NCT03153410
MultiTAA specific T cells (TAA-CTLs)	I	NCT03192462
Neoantigen peptide vaccine + Poly ICLC	I	NCT03956056
KRAS peptide vaccine + poly ICLC + anti-PD-1 (Nivolumab) + anti-CTLA4 (Ipilimumab)	I	NCT04117087
huCART-meso cells	I	NCT03323944
anti-MUC1 CAR T cells	I/II	NCT02587689
Gemcitabine + Nab-paclitaxel +/- anti-CTGF (Pamrevlumab)	III	NCT03941093
Gemcitabine + anti-PD-1 (Pembrolizumab) + FAK-inhibitor (Defactinib)	I	NCT02546531
FAK-inihbitor (Defactinib) + anti-PD-1 (Pembrolizumab)	I/II	NCT02758587
Anti-PD-1 (Pembrolizumab) + FAK-inhibitor (Defactinib)	II	NCT03727880
PEGylated Recombinant Human Hyaluronidase (PEGPH20) + anti-PD-1 (Pembrolizumab)	II	NCT03634332
PEGylated Recombinant Human Hyaluronidase (PEGPH20) + Gemcitabine + Nab-paclitaxel	N/A	NCT02921022

In short, the successful use of immunotherapy in PDAC patients remains an uphill battle. The research field should make optimal use of system-wide approaches integrating the different idiosyncrasies of the PDAC tumor microenvironment. Once immunotherapy is apt for PDAC patients, we believe that combinatorial treatment strategies will herald a new spark of light at the end of a long tunnel.
